# Estimation and correction of bias in network simulations based on respondent-driven sampling data

**DOI:** 10.1038/s41598-020-63269-0

**Published:** 2020-04-14

**Authors:** Lin Zhu, Nicolas A. Menzies, Jianing Wang, Benjamin P. Linas, Steven M. Goodreau, Joshua A. Salomon

**Affiliations:** 1 Department of Global Health and Population, Harvard T. H. Chan School of Public Health, Boston, MA USA; 2 Department of Medicine, School of Medicine, Stanford University, Stanford, CA USA; 3 Section of Infectious Disease, Department of Medicine, Boston Medical Center, Boston, MA USA; 4 Department of Epidemiology, Boston University School of Public Health, Boston, MA USA; 5 Department of Anthropology/Department of Epidemiology/Center for Studies in Demography and Ecology, University of Washington, Stanford University, Seattle, WA USA

**Keywords:** Disease prevention, Public health, Epidemiology, Applied mathematics, Statistics

## Abstract

Respondent-driven sampling (RDS) is widely used for collecting data on hard-to-reach populations, including information about the structure of the networks connecting the individuals. Characterizing network features can be important for designing and evaluating health programs, particularly those that involve infectious disease transmission. While the validity of population proportions estimated from RDS-based datasets has been well studied, little is known about potential biases in inference about network structure from RDS. We developed a mathematical and statistical platform to simulate network structures with exponential random graph models, and to mimic the data generation mechanisms produced by RDS. We used this framework to characterize biases in three important network statistics – density/mean degree, homophily, and transitivity. Generalized linear models were used to predict the network statistics of the original network from the network statistics of the sample network and observable sample design features. We found that RDS may introduce significant biases in the estimation of density/mean degree and transitivity, and may exaggerate homophily when preferential recruitment occurs. Adjustments to network-generating statistics derived from the prediction models could substantially improve validity of simulated networks in terms of density, and could reduce bias in replicating mean degree, homophily, and transitivity from the original network.

## Introduction

Respondent-driven sampling (RDS) is widely used for collecting data on hard-to-reach populations such as men who have sex with men, sex workers, and drug users^1,2^. In an RDS process, participants are asked about their partners and then provided a number of coupons to recruit their partners, with incentives or compensation for both recruiters and people recruited. The RDS approach was first proposed by Heckathorn^3^ to reduce biases introduced by snowball sampling and other chain-referral methods. By additionally providing incentives to partners being recruited instead of only to the recruiter, and encouraging participants to recruit their partners directly with a limited number of coupons rather than letting the investigators recruit from the name list provided by participants, RDS reduces four sources of bias: (1) sample dependence on the initial sample (seeds), (2) bias towards more cooperative subjects, (3) “masking” (protecting friends by not referring them), and (4) oversampling of people who have larger personal networks^3^. Several estimators have been proposed to derive population proportions from RDS samples, for example, to estimate the prevalence of HIV infection^4–6^. Estimators vary in terms of key assumptions, and studies have compared the robustness of various estimators and their underlying assumptions^7–11^.

Contact networks are becoming increasingly important in epidemiology, especially in studies of disease transmission and control. Contact networks are composed of individuals (nodes) and the relationships and interactions (ties/edges) among them. Data collected by chain-referral recruiting methods, including RDS and others, is used to estimate network structure and parameters for simulation models of pathogen transmission across a sexual or injection drug use network since these sampling methods provide information on both the individual and pair-wise (dyadic) levels^12–15^. Some studies use the sample network as a proxy for the full network and simulate disease transmission on this sample network^12^; others use information on network features from the sample network to reconstruct the whole network using statistical models such as exponential random graph models (ERGMs) or stochastic block model^13,15,16^.

Accurately capturing network structure is crucial, since it can play a significant role in disease transmission^16–21^. Three important features of network structure that impact disease transmission are: 1) density/mean degree, 2) homophily, and 3) transitivity^22,23^.

Density is defined as the proportion of all possible ties in the network that are actually present (Eq. (1)). Degree is the number of ties attached to a given node in the network, often summarized across the network as mean degree (the average number of ties per node, Eq. (2)). Density and degree are mathemacally related through network size (Eq. (3)); they determine the first-order level of connectivity shaping the overall extent and rate for the spread of infection. The calculations for density and mean degree are:

1 $$Den=\frac{T}{(\begin{array}{c}N\\ 2\end{array})}=\frac{2\times T}{N\times (N-1)}$$

2 $$MD=\frac{{\sum }_{i=1}^{N}{d}_{i}}{N}=\frac{2\times T}{N}.$$

3 $$\frac{MD}{Den}=N-1.$$

where Den is density, MD is mean degree, T is the total number of ties in the network, N is the network size/number of nodes in the network, and d_i_ is the degree of node i in the network.

Homophily describes the tendency to form ties between two nodes that have common attributes such as similar age, same gender, or same infection status. One statistic to represent homophily is the percentage of ties that are between “similar” nodes. Homophily can contribute to different levels of infections within different groups defined by the relevant attributes, especially in combination with other differences between those groups, e.g. different transmission probabilities, whether due to different contact frequency or use of prophylaxis such as condoms or needle exchange, in the case of HIV on sexual and injection networks, respectively.

Finally, transitivity describes the tendency to form ties between two nodes that are both connected to another node (“the friend of my friend is more likely to be my friend”). Higher transitivity means there will be more triangles (three nodes connected to each other) in the network than expected by chance. Instead of a simple count of triangles, however, a network statistic known as the “geometrically weighted edgewise shared partner” (GWESP) is commonly used to capture transitivity^24,25^. GWESP imposes a decreasing marginal effect of additional triangles on tie formation, beyond the first triangle closed by two nodes that share a partner, reflecting patterns often seen in empirical data. Like homophily, transitivity can represent a form of clustering, albeit one that is endogenous rather than formed on the basis of explicitly measured attributes. It thus has the potential to concentrate infection within clusters, while impeding its spread across clusters.

While the estimation of population proportions with RDS has been largely studied and improved, we are unaware of previous studies that have investigated the potential bias in network structure estimation caused by RDS; such bias is important because it can in turn bias epidemiological analyses and policy evaluations conducted using disease models that are overlaid on the network simulations. In this paper, we developed a mathematical and statistical platform to simulate different network structures with exponential random graph models, and to mimic the data generation mechanisms produced by RDS, as well as random sampling for comparison. We compared the three important network statistics– density/mean degree, homophily (in gender as an example), and transitivity – between the original networks and sample networks to identify the biases. Then we used generalized linear models to predict the network statistics of the original networks from the network statistics of the sample networks and sample design parameters.

## Results

### Density

**Figure 1:**
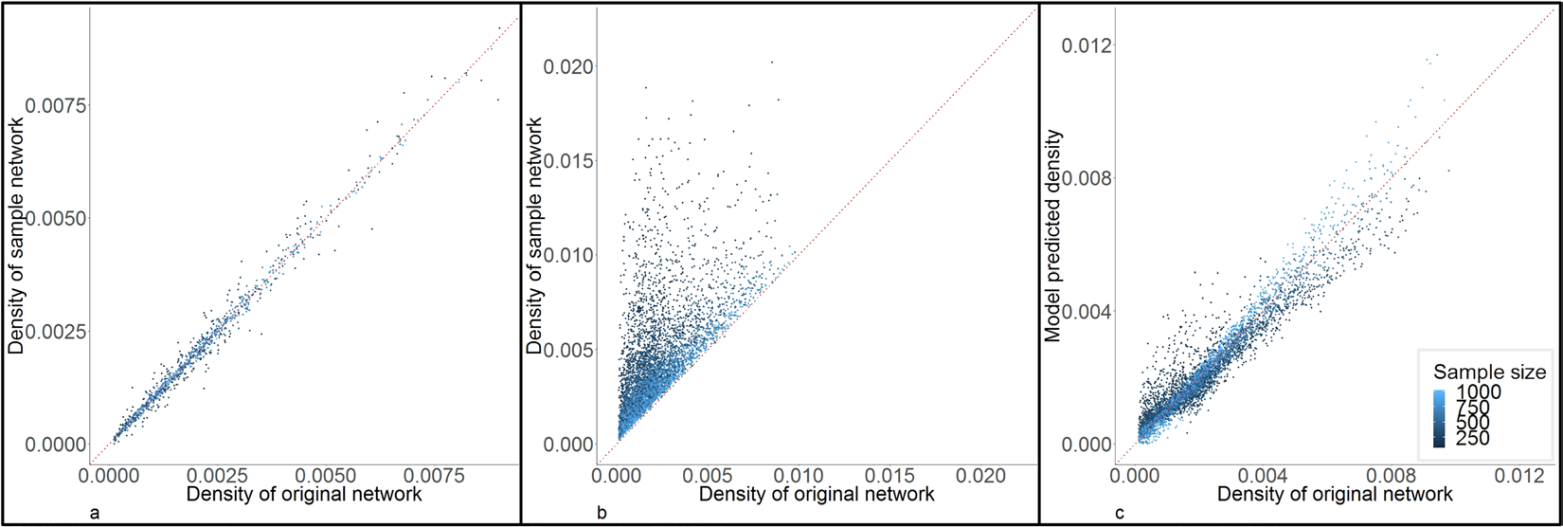
Estimation and correction of bias in density from RDS. Sub figures a and b are the comparison of density between original networks (x-axis) and sample networks (y-axis) in random samples (**a**) and RDS samples (**b**). Sub figure c is the comparison of density between original network (x-axis) and model correction from RDS (y-axis). The colors represent different sample sizes. The red dotted line is the diagnoal line to assist comparison.

Figure 1a,b shows the comparison of densities between the original and sample networks in random samples (**1a**) and RDS samples (**1b**). In random samples, the densities were close to the densities of the original networks, and the magnitude of the differences were negatively associated with sample size – as expected, the smaller sample sizes (dark points) have greater variances. The mean of the ratio of sample density to original density was 0.99 (SD = 0.13). On the other hand, most RDS samples over-estimated density; the magnitude of the bias was also negatively associated with sample size. In 10.10% and 1.82% of the RDS simulations, the density of the sample network was more than 5 and 10 times higher, respectively, than in the original network, and the mean of the ratio of sample density to original density was 2.60 (SD = 2.60).

Comparing predictive models for density, we found that linear models tended to minimize prediction errors. Based on our specified criteria, the selected linear model included 8 terms. Details on model selection are presented in the Supplement. The minimum root mean squared error (RMSE), mean absolute error (MAE), root mean squared percentage error (RMSPE), and mean absolute percentage error (MAPE) from all possible models of the four model families (linear, transformed, Poisson, and negative binomial) and from the null models are presented in Supplementary Table 1. Supplementary Fig. 1a shows the minimum RMSPEs by the number of terms in the linear models. The RMSPE and other error measures, and the adjusted R^2^ of the best model are presented in Table 1. The variables and coefficients of the best model are:

$$\begin{array}{rcl}{{\rm{D}}}_{0} & = & 1.024\times {10}^{-3}-1.592\times {10}^{-7}\times {{\rm{N}}}_{0}-2.296\times {10}^{-6}\times {N}_{s}-1.702\times {10}^{-3}\\  &  & \times {\rm{P}}+5.114\times {10}^{-1}\times {{\rm{D}}}_{{\rm{s}}}+4.14\times {10}^{-10}\times {{\rm{N}}}_{0}\times {{\rm{N}}}_{s}-7.167\\  &  & \times {10}^{-5}\times {{\rm{N}}}_{0}\times {{\rm{D}}}_{{\rm{s}}}+1.052\times {10}^{-3}\times {{\rm{N}}}_{{\rm{s}}}\times {{\rm{D}}}_{{\rm{s}}}-2.038\times {10}^{-1}\times {\rm{P}}\times {{\rm{D}}}_{{\rm{s}}}\end{array}$$

where D_0_ is density of the original network, N_0_ is the size of original network, N_s_ is the sample size, P is the proportion of the sample that was recruited from the chain-referral/partnerships, D_s_ is the density of the sample network. For an example of model correction for density, assume that we have an RDS sample network, the estimated original network size is 2000, the sample size is 300, the proportion of people recruited from partners is 0.3, and the density of the sample network is 0.004. Then the corrected density of the original network would be:

$$\begin{array}{c}1.024\times {10}^{-3}-1.592\times {10}^{-7}\times 2000-2.296\times {10}^{-6}\times 300-1.702\times {10}^{-3}\times 0.3+0.5114\\ \times \,0.004+4.14\times {10}^{-10}\times 2000\times 300-7.167\times {10}^{-5}\times 2000\times 0.004+1.052\\ \times \,{10}^{-3}\times 300\times 0.004-0.2038\times 0.3\times 0.004=2.24468\times {10}^{-3}.\end{array}$$

**Table 1:** Error measures of the best predictive models for network statistics.

Network statistics	RMSPE	Adjusted RMSPE^*^	RMSE	MAE	MAPE	Adjusted R^2^
Density	0.4143	0.4034	0.0005	0.0003	0.2164	0.9195
Mean degree	0.1056	NA	0.0769	0.0269	0.0182	0.9985
Homophily	5.2061	0.2122	0.0020	0.0338	0.3308	0.9761
Triangle density	17.1829	NA	0.0856	0.0673	1.4443	0.6264

^*^When there were negative predictions by linear models, the unadjusted values from the RDS samples were used instead of negative predictions.

**Figure 2:**
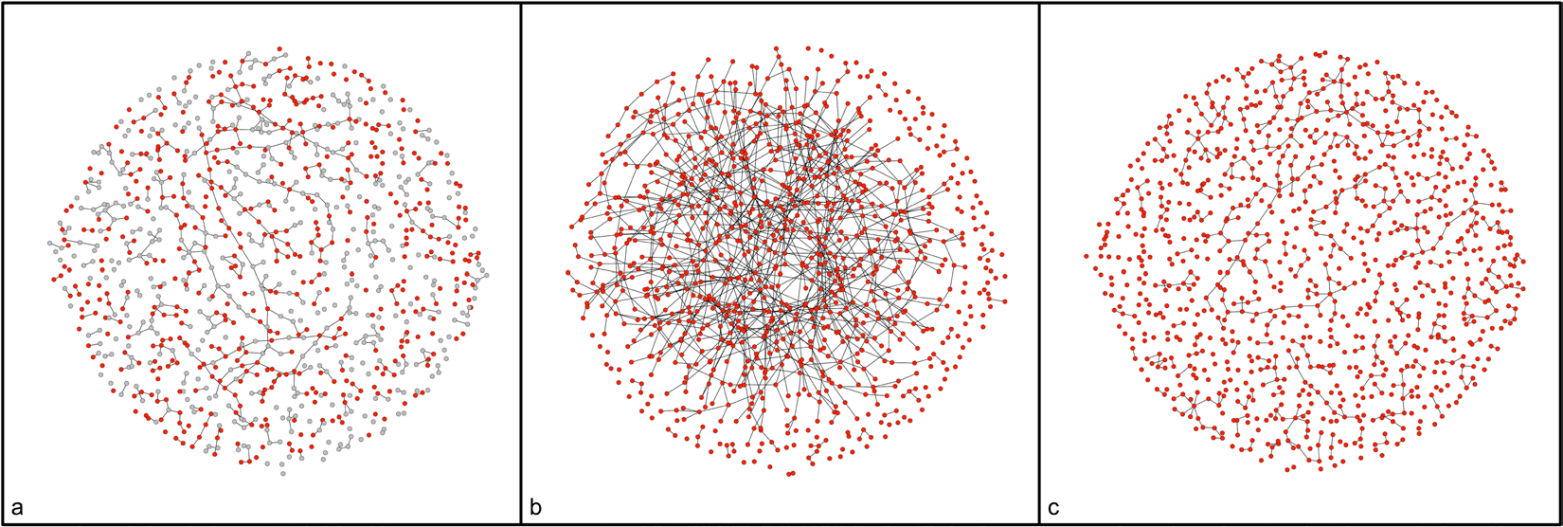
Example of simulated networks with and without density correction. Sub figure a shows the original network, nodes included in a simulated RDS sample from the original network is shown in red and nodes not included in the RDS sample is shown in grey, sub figure b is the network constructed directly with the density estimated from the RDS sample, and sub figure c shows the network constructed with the density corrected by our predictive model.

Figure 1c shows a comparison between the model-corrected densities and the densities of the original networks; the bias in density has been significantly reduced by the correction model. Figure 2 shows a network graph example illustrating the impact of our bias adjustment approach. The network constructed directly from the RDS sample density was visibly denser than the original network, and the network constructed from the model-corrected density appears much more similar to the original network.

### Mean degree

**Figure 3:**
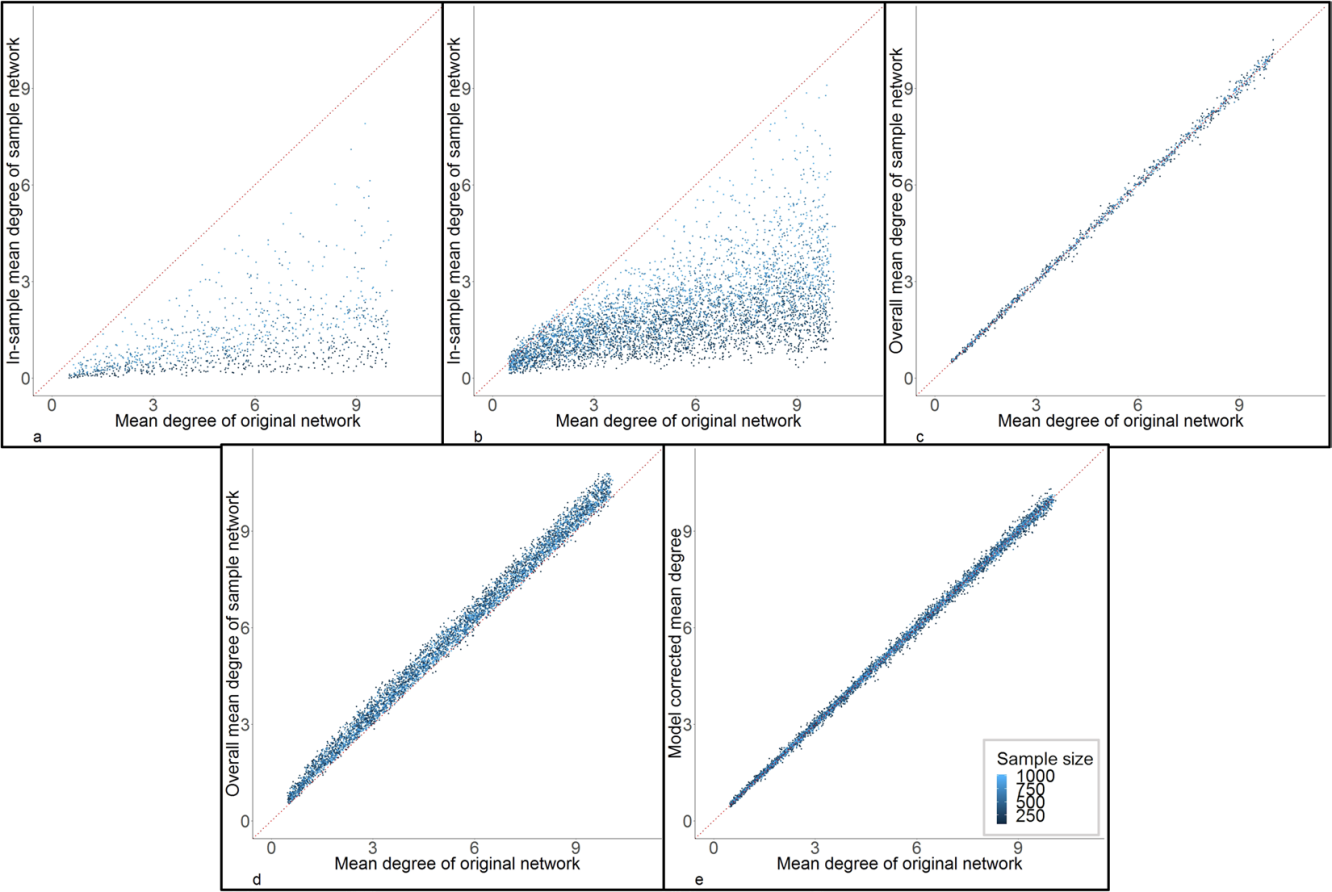
Estimation and correction of bias in mean degree from RDS. Sub figures a and b are the comparison of in-sample mean degree of sample networks (y-axis) and mean degree of original networks (x-axis) in random samples (**a**) and RDS samples (**b**). Sub figures c and d are the comparison of overall mean degree from sample networks (y-axis) and mean degree of original networks (x-axis) in random samples (**c**) and RDS samples (**d**). Sub figure e is the comparison of mean degree between original network (x-axis) and model correction from RDS (y-axis). The colors represent different sample sizes. The red dotted line is the diagnoal line to assist comparison.

**Figure 4:**
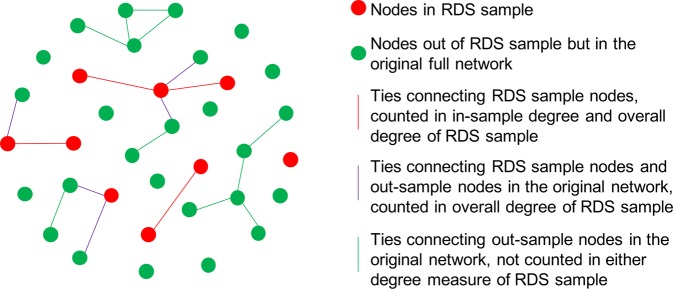
In-sample degree and overall degree definitions. This schematic distinguishes different types of nodes and ties based on their inclusion in or exclusion from the RDS sample, and illustrates the basis for calculating in-sample degree and overall degree.

Figure 3a,b shows the comparison of in-sample mean degree (defined in Methods section and Fig. 4) of the random samples (**3a**) and RDS samples (**3b**) to the mean degree of the original networks. In both random samples and RDS samples, the in-sample mean degrees were smaller than the mean degrees of the original networks. The error was also negatively associated with sample size. Figure 3c,d shows the comparison of overall mean degree (defined in Methods section and Fig. 4) of the random samples (**3c**) and RDS samples (**3d**) to the mean degree of original networks. The overall mean degree of random samples provided good approximations of the mean degree of the original networks. The overall mean degree of RDS samples overestimated the mean degree of the original networks.

Again, linear prediction models produced the minimum errors; the selection information is presented in Supplementary Table 2 and Supplementary Fig. 1b. The RMSPE and other error measures and the adjusted R^2^ of the best model are presented in Table 1. The variables and coefficients of the best model are:

$$\begin{array}{c}{{\rm{MD}}}_{0}=-\,6.791\times {10}^{-2}+2.445\times {10}^{-5}\times {{\rm{N}}}_{0}+1.525\times {10}^{-4}\times {{\rm{N}}}_{{\rm{s}}}-8.491\times {10}^{-1}\\ \,\times \,{\rm{P}}+9.992\times {10}^{-1}\times {{\rm{MD}}}_{{\rm{s}}}-4.961\times {10}^{-8}\times {{\rm{N}}}_{0}\times {{\rm{N}}}_{{\rm{s}}}\\ \,-5.282\times {10}^{-5}\times {{\rm{N}}}_{0}\times {\rm{P}}+2.504\times \,{10}^{-4}\times {{\rm{N}}}_{{\rm{s}}}\times {\rm{P}}\end{array}$$

where MD_0_ is mean degree of the original network, N_0_ is the size of original network, N_s_ is the sample size, P is the proportion of the sample that was recruited from the chain-referral/partnerships, MD_s_ is the overall mean degree of the sample network. Figure 3e shows the comparison between the model-corrected mean degree and the mean degree of the original networks; the bias in mean degree has been reduced by the correction model.

### Homophily

**Figure 5:**
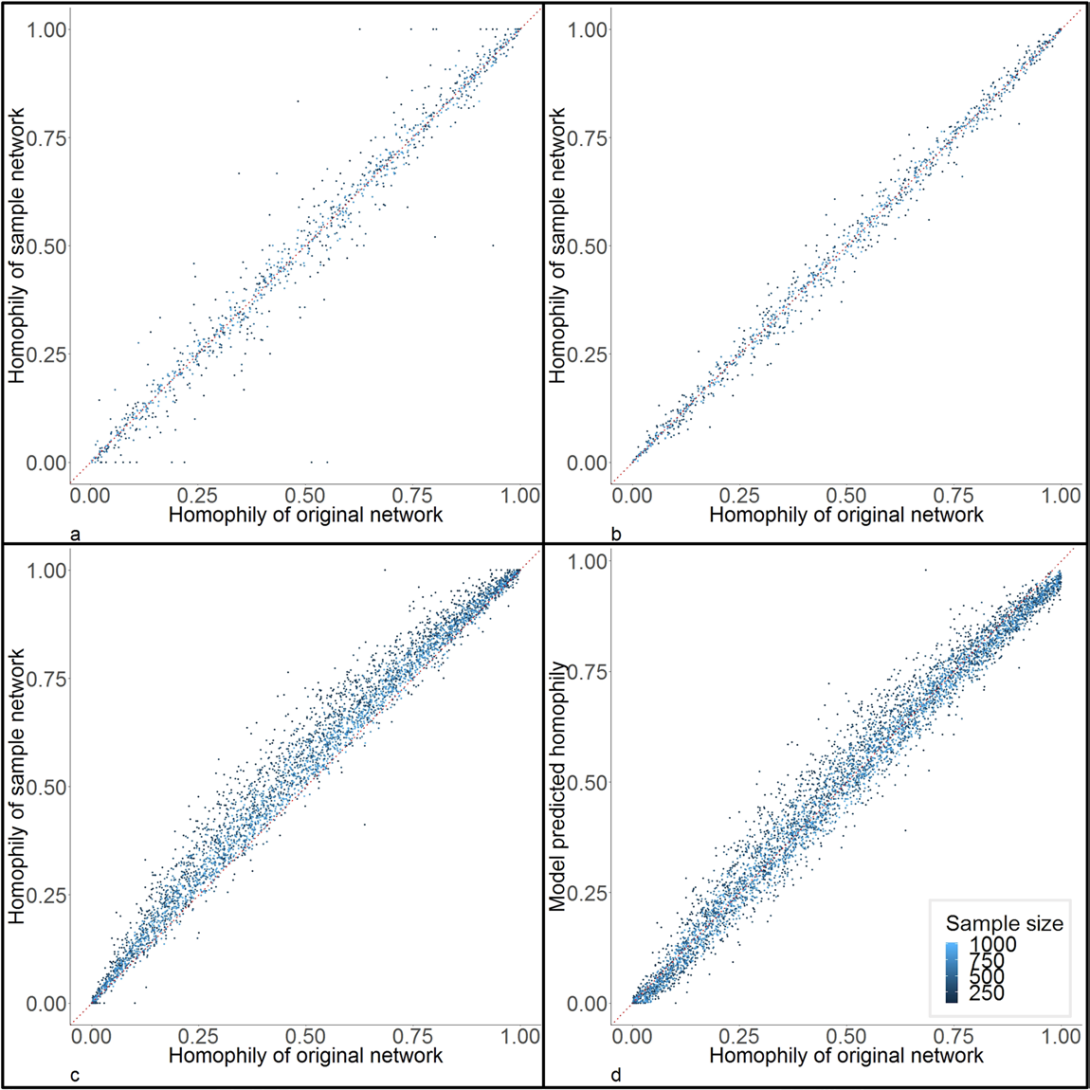
Estimation and correction of bias in homophily (in gender) from RDS. Sub figures a, b, and c are the comparison of homophily between original networks (x-axis) and sample networks (y-axis) in random samples (**a**), RDS samples without preferential recruitmen t (**b**), and RDS samples with preferential recruitment (**c**). Sub figure d is the comparison of homophily between original network (x-axis) and model correction from RDS (y-axis). The colors represent different sample sizes. The red dotted line is the diagnoal line to assist comparison.

Figure 5a–c shows the comparison of gender homophily between the original and sample networks, in random samples (**5a**), RDS samples with non-preferential recruitment (**5b**), and RDS samples with preferential recruitment (**5c**). As described in detail in the Methods section, preferential recruitment means subjects are more likely to give coupon to their partners with the same gender. Random sampling and RDS with non-preferential recruitment did not bias homophily overall, although in random samples with small sample size, the variance was large. Most RDS with preferential recruitment over-estimated homophily, but the bias was relatively small. The means of the ratio of sample homophily to original homophily for random samples, RDS with non-preferential recruitment, and RDS with preferential recruitment were 0.99 (SD = 0.29), 1.00 (SD = 0.14), and 1.15 (SD = 0.23), respectively.

Again, linear models minimized prediction errors; the selection information is presented in Supplementary Table 3 and Supplementary Fig. 1c. The RMSPE and other error measures and the adjusted R^2^ of the best model are presented in Table 1. The variables and coefficients of the best model are:

$$\begin{array}{c}{{\rm{H}}}_{0}=-\,3.232\times {10}^{-3}-1.605\times {10}^{-3}\times {\rm{C}}-9.2769\times {10}^{-2}\times {\rm{P}}+9.89396\times {10}^{-1}\\ \,\times \,{{\rm{H}}}_{{\rm{s}}}-9.12684\times {10}^{-1}\times {{\rm{D}}}_{{\rm{s}}}+1.9231\times {10}^{-2}\times {\rm{C}}\times {\rm{P}}\\ \,-4.51862\times {10}^{-1}\times {\rm{C}}\times {{\rm{D}}}_{{\rm{s}}}\end{array}$$

where H_0_ is homophily (in gender) of the original network, C is number of coupons, P is the proportion of the sample that was recruited from the chain-referral/partnerships, H_s_ is the homophily (in gender) of the sample network, D_s_ is the density of the sample network. Figure 5d shows the comparison between the model-corrected homophily and the homophily of the original networks; the bias in homophily has been reduced by the correction model.

### Transitivity

**Figure 6:**
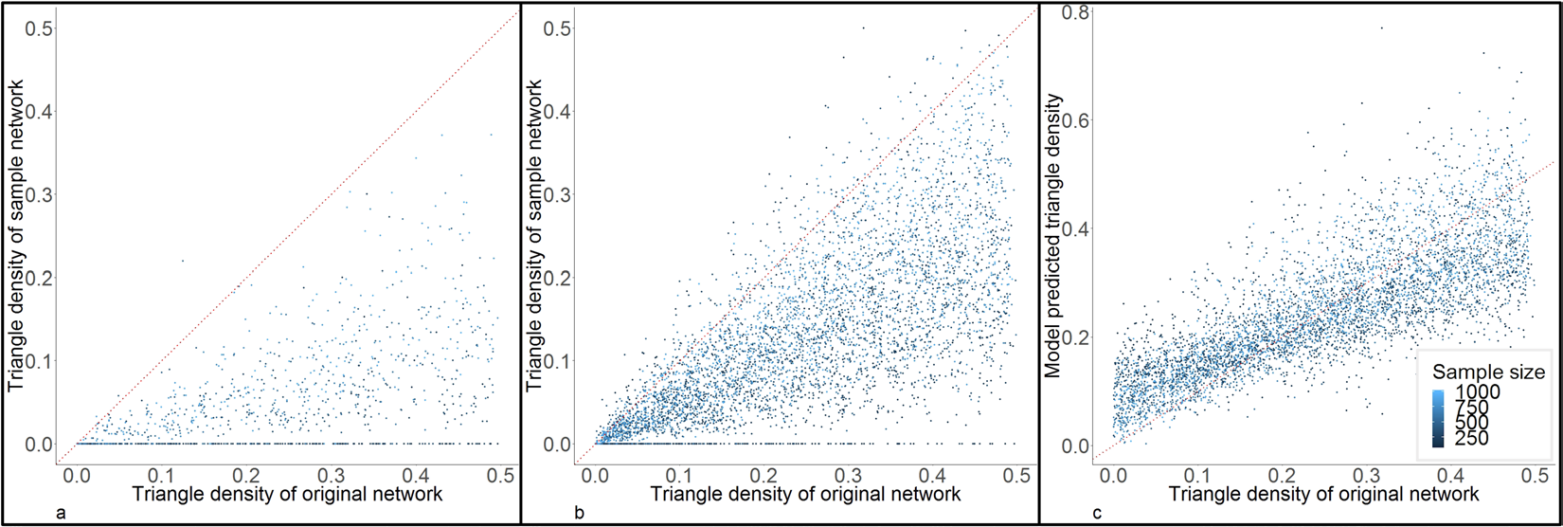
Estimation and correction of bias in transitivity (triangle density) from RDS. Sub figures a and b are the comparison of triangle density between original networks (x-axis) and sample networks (y-axis) in random samples (**a**) and RDS samples (**b**). Sub figure c is the comparison of triangle density between original network (x-axis) and model correction from RDS (y-axis). The colors represent different sample sizes. The red dotted line is the diagnoal line to assist comparison.

Figure 6a,b shows the comparison of the triangle density (defined in Methods section) between the original and sample networks in random samples (**6a**) and RDS samples (**6b**). In the majority of the samples, both random sampling and RDS under-estimated triangle density. In a number of extreme cases (30% in random samples, 7.36% in RDS samples), the sampling process did not capture transitivity in the network at all, i.e. the sampe had a triangle density of 0; the sample sizes for these cases were small.

Once again, linear models produced the minimum prediction errors, with the selection information presented in Supplementary Table 4 and Supplementary Fig. 1d. The RMSPE and other error measures and the adjusted R^2^ of the best model are presented in Table 1. The variables and coefficients of the best model are:

$$\begin{array}{c}{{\rm{TD}}}_{0}=1.774\times {10}^{-2}+3.392\times {10}^{-5}\times {{\rm{N}}}_{0}-1.001\times {10}^{-2}\times {\rm{C}}-1.067\times {10}^{-2}\\ \,\times {\rm{P}}\times {{\rm{TD}}}_{{\rm{s}}}+16.64\times {{\rm{D}}}_{{\rm{s}}}-4.255\times {10}^{-5}\times {{\rm{N}}}_{0}\times {\rm{P}}+2.346\times {10}^{-3}\\ \,\times {{\rm{N}}}_{0}\times {{\rm{D}}}_{{\rm{s}}}-2.539\times {10}^{-1}\times {\rm{C}}\,\ast \,{{\rm{D}}}_{{\rm{s}}}-16.61\times {\rm{P}}\times {{\rm{D}}}_{{\rm{s}}}\end{array}$$

where TD_0_ is the triangle density of the original network, N_0_ is the size of original network, C is number of coupons, P is the proportion of the sample that was recruited from the chain-referral/partnerships, TD_s_ is triangle density of the sample network, D_s_ is the density of the sample network. Figure 6c shows the comparison between the model-corrected triangle densities and the triangle densities of the original networks; the bias in triangle density has been largely reduced by the correction model, although residual error still exists, especially when sample size was small.

## Discussion

Network models have been developed in recent years to represent the underlying heterogeneous contact networks among the population, and in some cases used to simulate disease transmission dynamics. RDS provides a means to obtain information on the network structure, and many network models are using chain-referral samples to represent or reconstruct their underlying disease transmission networks, but none have considered the potential biases in constructing networks based on the RDS data. In this paper, we assessed biases that can arise through RDS methods in three major network statistics – density/mean degree, homophily, and transitivity – and proposed an approach to correct the biases.

Our study demonstrates that RDS tends to over-estimate network density. In addition, in-sample mean degree in both random and RDS sample networks under-estimates the mean degree, and the overall mean degree from RDS samples over-estimates the mean degree. Since the overall mean degree is calculated from the true number of ties of each node (Fig. 4), the result of higher overall mean degree in the RDS samples than in the original networks also implies that higher-degree nodes were more likely to be recruited in RDS. It is important to note that we did not directly impose a higher probability of being recruited given higher degree in our simulation; rather, this oversampling is induced by the RDS approach. RDS was designed to reduce the bias of oversampling higher-degree nodes, but in our analysis, it did not eliminate this bias. This bias is closely associated with the probability of recruiting a random person in the network versus a partner (as a sampling design factor) or the proportion of the sample that is recruited from partner-referral (as a parameter that can be analyzed from the RDS dataset). In many RDS sampling schemes, people are asked to list and recruit their partners, and this will result in an under-representation of isolated people who do not have partners. In other RDS studies, partnership may not be required as a recruitment criterion, so there may be less recruitment from partner-referral. In this latter case, the estimation of mean degree would be more similar to that in random samples, which would have less bias. Nevertheless, as long as the sampling process involves partner recruitment, this bias cannot be completely eliminated. Our correction model for mean degree includes as a predictor the proportion of the sample that was recruited from partner-referral, and examining this outcome directly may provide insight about the magnitude of bias in mean degree.

There are debates about whether to preserve network density or to preserve mean degree when using sample network data to expand and re-construct the original network^26^. Our study reveals that preserving either the density or the mean degree of the RDS sample leads to bias. Plots of the networks constructed with the un-corrected density and corrected density clearly illustrate the dramatic difference in network structures. Simulation of pathogen transmission across a network largely depends on the number of partnerships in the network; for example, with the same network size and contact frequency, networks with higher density will have more transmission events. Therefore, studies that use unadjusted estimates of density or mean degree derived from RDS can lead to over-estimation of incidence. On the other hand, if the contact frequency or transmission probability per contact is unknown and requires model calibration, then with the same target incidence, networks with higher density/mean degree can lead to calibration results of lower contact frequency or transmission probability. Increasing the sample size will reduce the magnitude of bias in density, but because the costs of data collection increase with sample size, model correction may be an attractive alternative in many cases. Our prediction model significantly improved the density and mean degree estimation from the RDS sample.

Homophily is another important network statistic that can impact the disease transmission pattern between different groups. We used gender as an example, but other examples may include age, or preference in personal network size (i.e. persons that prefer small personal networks may be likely to form partnerships with other persons who also prefer small personal networks). Homophily in age and gender might lead to disproportional disease transmission in different age groups or in different genders. Homophily in personal network size allows relatively more stable partnerships in networks: people who prefer smaller personal networks are less likely to connect to the bigger components in the networks and have lower probability of being infected. Our study has shown that although random sampling and RDS with non-preferential recruitment had random error in homophily, it did not bias the homophily overall. Thus, if there is no evidence that participants are more likely to recruit partners that have the same/similar attribute as them, homophily estimation from the sample network can be directly used to construct the original network. However, if preferential recruiting behavior does exist, it tends to be exaggerated by RDS and correction is recommended.

Higher transitivity in a network shortens the transmission path: if A is connected to both B and C, and when transitivity is low, B and C are not more likely to be connected, but when transitivity is high, B and C are more likely to be connected. Thus, with higher transitivity, pathogens can go from B to C directly without passing through A. Networks with higher transitivity will have greater local clustering that is not tied to specific exogenous attributes, which can speed up transmission within the cluster, but also (for a fixed density) keep the pathogen constrained within cluster. In networks with lower transitivity on the other hand, pathogens can spread more slowly but more widely. In both random and RDS sample networks, triangle density was largely under-estimated. Our correction model has reduced the bias, but substantial residual error remains, especially with small samples^27,28^.

Based on the comparisons of random sampling and RDS in this study (Figs. 1, 3, 5 and 6), random sampling did not bias density, overall mean degree, and homophily, but it caused more severe underestimation of transitivity than RDS. Adjusting the sampling design to increasing recruitment of eligible acquaintances instead of partners may increase the proportion of the final sample that resembles a “random” sample, and hence reduce bias in density, overall mean degree, and homophily, but it might exacerbate the underestimation of transitivity.

In this paper we explored three important network statistics. However, these are not the only ones that may have an impact on disease transmission networks; for example, the proportion of individuals with concurrent partners has received considerable focus as a driver of generalized HIV epidemics on sexual networks^29,30^. We explored homophily on an attribute—gender—which is categorical, and which took two values in our data; however, relational mixing can occur on continuous variables (e.g. age) or those with different numbers of categories (e.g. race) too. In addition, in some networks, there can be imbalanced mixing, for example, different extent of homophily in different subgroups or asymmetric age mixing across genders.

There are multiple variations of RDS, which vary in the classes of relevant parameters. We focused on the three basic sampling design parameters that likely could be obtained from any RDS (sample size, number of coupons provided, and proportion of participants that were recruited from chain-referral) in our models to correct the biases. However, if additional information can be collected during the sampling process (for example, the partnerships between the partners that a participant has named), this information might improve the estimation of transitivity. We also tried to allow flexibility of the sampling process to mimic different RDS designs; for example, coupons could be given to a “random” person in the original network instead of only to partners, which could happen when a participant who has a limited number of partners gives out more coupons than (s)he has partners in order to gain more incentive payments that are linked to referring new individuals to the study. It can also happen when a coupon is given to an acquaintance who does not have the required type of partnership with the recruiter; e.g. in studies of injection equipment sharing networks, the respondent could give the coupon to an acquaintance who also injects drugs but with whom (s)he does not share equipment. While our study did not attempt to focus on detailed coupon distribution behaviors, such a focus merits further consideration in future work. Examples of possible avenues for further inquiry include, for example, whether people with higher degree may be more motivated to distribute coupons, or more willing to accept coupons and participate in a study; or whether people may be more likely to recruit partners than non-partners if partnership is not a requirement. Current understanding of these psychological and behavioral factors remains very limited. If such factors are prominent, they may increase bias in density, mean degree, and homophily, while potentially decreasing bias in transitivity.

An important limitation in our analysis is that we did not specify the degree distribution in ERGMs for simulating the original networks, and did not model homophily in any attributes that would partition the entire population into several major clusters. Networks using other simulation mechanisms such as preferential attachment models and stochastic block models may produce distinct patterns in networks. Since we could not include all possible types of network features in this study, the correction models we report may not generalize broadly beyond the sampling mechanisms and parameter ranges used in our study.

While our simulation study allowed us to quantify biases arising from estimation of network features from an RDS sample, validation of our approach against strictly empirical data remains an elusive goal. Such an empirical validation would require data that describe both the full original network and the RDS sample from it, yet we are not aware of any datasets that are suitable for this purpose. For disease transmission networks such as men who have sex with men and people who inject drugs, acquiring the full underlying network is difficult, if not impossible, since these populations are usually hidden or hard-to-reach due to stigma and other factors. While there might be limited possibility to assess goodness-of-fit without knowledge of the underlying network, such assessment would be confined to density, overall mean degree, and homophily, and would require that the sample meet additional assumptions. For example, if a subset of participants are known to have joined the sample as ‘random’ recruits rather than as partners of current members of the sample, this subsample may supply unbiased estimates of density, mean degree and homophily in the full underlying network, and may therefore serve as a comparator to assess the goodness of fit of the correction models. However, because this subsample would have a smaller overall sample size (compared to the full sample), there will be greater sampling error in any estimated quantity. Moreover, such a strategy depends on having a subsample whose recruitment was truly random, which may not be compatible with many RDS designs.

In short - we could not examine all possible types of networks and network statistics in this one study. Nevertheless, our results incorporating three highly used statistics across a range of typical disease transmission networks illustrate how RDS may bias the estimation of network structures, and we present an approach to adjusting for these biases based on features and outcomes that are commonly observed in RDS-based network studies.

## Conclusion

RDS can introduce significant bias in estimation of network density, mean degree, and transitivity. Homophily tends to be slightly exaggerated when preferential recruitment exists. Adjustments to network generating statistics derived from the prediction models can substantially improve validity of simulated networks in terms of density, and can reduce bias in replicating mean degree, homophily, and transitivity from the original network.

## Methods

### Overview

We developed a platform to simulate network structures and the data generation mechanisms produced by RDS, as well as random sampling for comparison. In each simulation, we first generated an original network with a variety of different values of network statistics. Random sampling or RDS was then simulated on this original network to generate a sample network, based on values of an additional set of parameters reflecting different sample design factors. We ran 1000 simulations for random sampling to estimate the biases, and 5000 simulations for RDS to both estimate and correct the biases. A different vector of network statistics and sampling parameters was drawn for each simulation. We then conducted two types of analyses. First, we compared each class of network statistics between the sample network and the original network to identify biases. Second, we fitted predictive models to correct these biases by allowing estimation of the true statistics pertaining to the original network from the observed statistics of the sample network and known sample design features.

### Network statistics

The original full networks were generated using ERGM with the Statnet package in R^31^. Uniform distributions were used to draw the varying parameters relating to network statistics in the generation of multiple simulated networks, and the ranges for the network statistics are summarized in Table 2. Nodes were distinguished by gender, with all networks having equal numbers of males and females. Gender homophily was defined as the proportion of ties between pairs with the same gender. Transitivity was represented as the proportion of ties in at least one triangle, which we call triangle density. This builds off the common use of the ERGM term GWESP with a decay parameter of 0, which counts the number of ties in at least one triangle; however, we normalize this to a proportion to ease comparison across networks of different sizes. We found a linear relationship between the number of triangles and the GWESP (decay = 0) statistic in our simulation networks, confirming that GWESP (decay = 0) was a good summary statistic for overall transitivity. For the sample network, degree for each node was calculated in two ways: 1) in-sample degree of the sample–only ties connected to nodes that were also within the sample were counted; 2) overall degree of the sample–all ties between members of the sample and any other node in the full original network were counted, whether or not the other node was also present in the sample (Fig. 4). Although overall degree cannot be observed directly from the graph of the sample network, RDS participants are often asked about all of their partnerships (not limited to partnerships with the other participants in the sample), which provides an indicator of overall degree (to the extent that reported measures are unbiased).

**Table 2:** Ranges of sampling parameters and network features in the simulations.

Parameters	Range	Reference
**Network statistics of original network**		
Network size	1000–5000	Assumed
Mean degree	0.5–10	^39–42^
Gender homophily	0–1	Full range
Triangle density	0–0.5	Assumed
**Sampling design parameters**		
Sample size	100–1000	^15,43^
Number of coupons provided	1–5	^15,43^
Probability of giving coupon to random person	0.1–0.9	Full range
Odds of preferential recruitment	2–5	Assumed

### Simulation of the sampling process

The sampling design parameters were drawn from uniform distributions with ranges summarized in Table 2. In the random sampling approach, a number of nodes were randomly selected from the original network. In the RDS approach, the sampling process began with one seed (initial sample), followed by chain-referral recruitment (described in detail in the next paragraph). Additional seeds were recruited when all the referral chains had ended but the desired sample size had not been met. The seeds were randomly selected from the original network instead of being selected proportionally to nodal degree because 1) it has been suggested that the composition of an RDS sample is not dependent on the selection of seeds^3,32–34^; and 2) most empirical RDS studies were based on a convenience sample of seeds, in which the correlation between the selection probability and nodal degree or other attributes was not clear^34,35^. Prior to finalizing our simulation design, we ran a number of test simulations recruiting seeds with probability proportional to nodal degree, and confirmed that this choice did not qualitatively affect the results, so we opted for the simpler approach here.

After a simulated person was recruited into the sample, (s)he was provided the number of coupons determined by the coupon parameter, but (s)he could choose not to give out all the coupons^36^. The number of coupons a person intended to give out was determined by a discrete uniform distribution between 0 and the number of coupons provided. For each coupon intended to be given out, the recruiter had a probability of giving it to a random person in the network instead of a partner.

In the simulation, if a coupon was not given to a random person, then the recruiter gave it to one of his/her partners. If there were more coupons left than the number of partners (s)he had, the extra coupons were discarded. The probability of recruiting each partner could be equal (non-preferential recruitment) or based on preferential recruitment of partners sharing the same gender^37^. Among the 5000 RDS simulations used to examine homophily, 1000 assumed non-preferential recruitments and 4000 assumed preferential recruitment. The probability of recruiting each partner was determined by a parameter specifying the odds of preferential recruitment, which is the ratio of the probability of recruiting a partner with the same gender to the probability of recruiting a partner with the different gender.

To mimic a typical RDS, in the simulation, participants were not allowed to recruit the person who directly recruited them. However, participants could give coupons to persons who were already in the sample, in which case those persons would not be put into the sample again. Each new recruitment was added to the sample, and each person in the sample could recruit new persons until the sample size was met.

### Predictive models

Generalized linear models were used to predict the density, mean degree, homophily, and transitivity in the original network from the RDS parameters and RDS sample network statistics. The variables used in all models included: size of original network, number of coupons, sample size, and proportion of the sample that was recruited from the chain-referral/partnerships. Density of the sample network, overall mean degree of the sample network, homophily of the sample network, and triangle density of the sample network were included in models predicting each of these network statistics for the full original networks. Density of sample network was also included in models predicting homophily and triangle density. Since in-sample mean degree and sample density are mathematically linked (Eq. (3) and Fig. 4), we only used sample density to predict original density, and then overall sample mean degree to predict original mean degree.

We conducted a model selection process by considering all possible combinations of the main effects and two-way interaction terms. A linear model, transformed linear model (log transformation for density and mean degree, logit transformation for homophily and triangle density), Poisson model, and negative binomial model were compared. For the Poisson and negative binomial models, the outcome variables were transformed to counts (number of edges for density and mean degree, number of edges between the same gender for homophily, and number of edges in at least one triangle for triangle density), and offset terms which were the logarithm of the denominators for the transformation (maximum number of edges in a given network for modeling density, network size divided by two for modeling mean degree, number of edges for modeling homophily and triangle density) were applied. For example, the number of edges was used as the outcome variable instead of density in Poisson regression, and the model included the offset term of the logarithm of the maximum number of possible edges in the network. As the linear model can produce predictions that are outside of the range of the quantities being predicted (e.g. negative values for density), we modified the results to provide no adjustment in these instances, and instead returned the unadjusted survey value.

Ten-fold cross validation^38^ was used for model selection. To implement this, the data were divided into 10 equal-sized groups. For each of these groups, we predicted quantities of interest from a model estimated using the remaining groups. Error measures were calculated by comparing observed values to these out-of-sample predictions. This procedure was conducted for all 10 groups. We used root mean squared percentage error (RMSPE) between the original network statistics and model predicted network statistics as the primary goodness of fit measure for model selection. We also calculated root mean squared error (RMSE), mean absolute error (MAE), and mean absolute percentage error (MAPE) for each model to enable more detailed comparison. In the relative error measures RMSPE and MAPE, 0.0001 was added to the denominator (observed network statistics of the original network) to avoid zero values. We plotted the minimum error that models with each number of model terms could have, and then selected the best model as the model that had the smallest number of model terms within the subset of models that had a RMSPE that fell within 1% of the minimum RMPSE across all possible models.

## Supplementary information

Supplementary information.

## Data Availability

The datasets generated during and analyzed during the current study are available from the corresponding author on reasonable request.
