# Supplementary material for: Estimation and correction of bias in network simulations based on respondent-driven sampling data
Source: Sci Rep. 2020 Apr 14;10:6348. doi: 10.1038/s41598-020-63269-0 (PMC7156755; doi:10.1038/s41598-020-63269-0)
Supplement: Supplementary file 1 — Supplementary information. [file 41598_2020_63269_MOESM1_ESM.pdf]

Estimation and correction of bias in network simulations based on respondent-driven sampling data

Supplement Materials

Lin Zhu, Nicolas A. Menzies, Jianing Wang, Benjamin P. Linas, Steven M. Goodreau,

Joshua A. Salomon

Table 1 Error measures of predictive models for network density

Model Family	Model terms	RMSE	MAE	RMSPE	MAPE
Linear models	Null model with only intercept	0.0017	0.0012	1.7233	0.9873
	Null model with intercept and sample density	0.0013	0.0009	1.0603	0.6017
	Model producing the minimum error	0.0005	0.0003	0.4115	0.2164
Linear models with log transformation	Null model with only intercept	0.0017	0.0012	1.7233	0.9873
	Null model with intercept and sample density	0.0014	0.0010	1.3273	0.7799
	Model producing the minimum error	0.0006	0.0005	0.6566	0.3808
Poisson models	Null model with only intercept	0.0018	0.0012	1.1438	0.7121
	Null model with intercept and sample density	0.0015	0.0010	0.9090	0.5900
	Model producing the minimum error	0.0009	0.0006	0.5247	0.3113
Negative binomial models	Null model with only intercept	0.0017	0.0012	1.7229	0.9871
	Null model with intercept and sample density	0.0025	0.0012	1.4854	0.7094
	Model producing the minimum error	0.0013	0.0007	0.5317	0.3062

Table 2 Error measures of predictive models for network mean degree

Model Family	Model terms	RMSE	MAE	RMSPE	MAPE
Linear models	Null model with only intercept	2.7265	2.3577	1.7631	0.9153
	Null model with intercept and sample mean degree	0.2245	0.1841	0.0748	0.0500
	Model producing the minimum error	0.1056	0.0769	0.0268	0.0182
Linear models with log transformation	Null model with only intercept	2.7265	2.3577	1.7631	0.9153
	Null model with intercept and sample mean degree	0.6317	0.5177	0.5011	0.2268
	Model producing the minimum error	0.5875	0.4857	0.4665	0.2136
Poisson models	Null model with only intercept	2.7264	2.3577	1.7600	0.9140
	Null model with intercept and sample mean degree	0.6980	0.5768	0.3970	0.1980
	Model producing the minimum error	5.9660	5.3080	0.9987	0.9987
Negative binomial models	Null model with only intercept	5.9669	5.3079	0.9988	0.9988
	Null model with intercept and sample mean degree	5.9664	5.3083	0.9993	0.9993
	Model producing the minimum error	5.9660	5.3080	0.9987	0.9987

Table 3 Error measures of predictive models for network homophily

Model Family	Model terms	RMSE	MAE	RMSPE	MAPE
Linear models	Null model with only intercept	0.0825	0.2485	124.8853	5.8666
	Null model with intercept and sample homophily	0.0021	0.0350	7.4709	0.3920
	Model producing the minimum error	0.0018	0.0316	5.2060	0.3174
Binomial models with logit transformation	Null model with only intercept	0.0825	0.2485	124.8853	5.8666
	Null model with intercept and sample homophily	0.0029	0.0419	14.6215	0.6700
	Model producing the minimum error	0.0025	0.0394	13.9500	0.6479
Poisson models	Null model with only intercept	0.0825	0.2485	124.8807	5.8688
	Null model with intercept and sample homophily	0.0059	0.0647	30.6947	1.3908
	Model producing the minimum error	0.0056	0.0612	30.1100	1.3790
Negative binomial models	Null model with only intercept	0.0825	0.2485	124.8840	5.8665
	Null model with intercept and sample homophily	0.0165	0.0948	23.3530	1.1058
	Model producing the minimum error	0.0162	0.0922	22.5000	1.0800

Table 4 Error measures of predictive models for network triangle density

Model Family	Model terms	RMSE	MAE	RMSPE	MAPE
Linear models	Null model with only intercept	0.1400	0.1206	77.9709	4.9550
	Null model with intercept and sample transitivity	0.0968	0.0791	36.2724	2.3881
	Model producing the minimum error	0.0802	0.0616	17.1800	1.4300
Binomial models with logit transformation	Null model with only intercept	0.1849	0.1515	72.5622	4.6158
	Null model with intercept and sample transitivity	0.1019	0.0835	42.5574	2.7434
	Model producing the minimum error	0.0873	0.0682	29.3800	2.0970
Poisson models	Null model with only intercept	0.1400	0.1207	79.3953	5.0463
	Null model with intercept and sample transitivity	0.1336	0.1113	68.6957	4.3863
	Model producing the minimum error	0.1180	0.0795	30.5200	2.1970
Negative binomial models	Null model with only intercept	0.1400	0.1206	77.9758	4.9553
	Null model with intercept and sample transitivity	0.1469	0.1134	63.8433	4.0928
	Model producing the minimum error	0.1302	0.0881	24.6300	1.8420

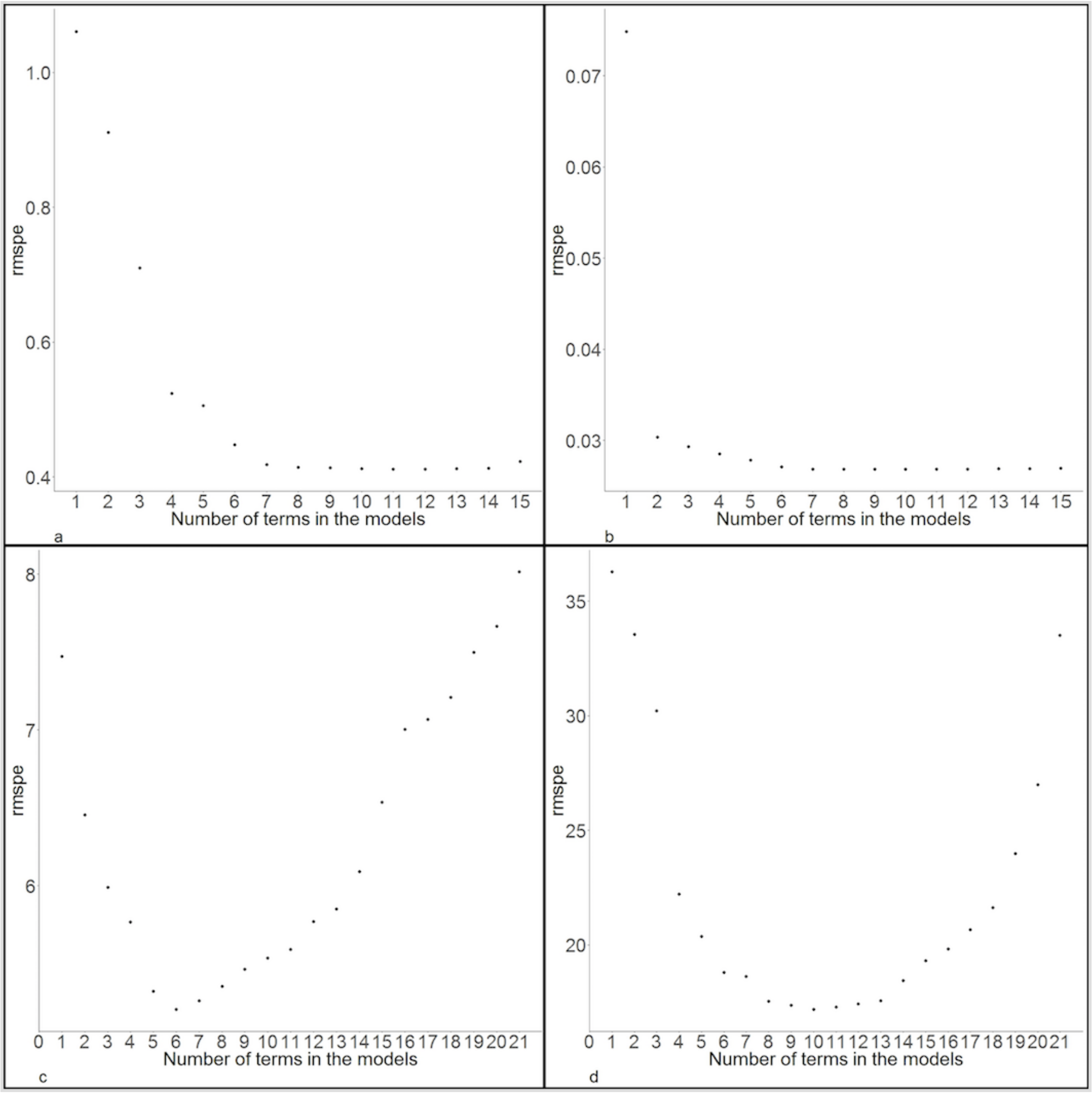

Figure 1 Minimum RMSPE by the number of terms in the models. The y-axis represents the minimum RMSPE of models that have certain number of terms. Sub figure a is for density, b is for mean degree, c is for homophily, and d and for triangle density.
